# A Case of Sinus of Valsalva Aneurysm Associated With a Single Coronary Artery Successfully Treated by Bentall Surgery

**DOI:** 10.1093/icvts/ivag098

**Published:** 2026-04-06

**Authors:** Kotaro Mukasa, Shinichiro Abe, Yasunori Yakita, Soichi Asano

**Affiliations:** Department of Cardiovascular Surgery, Chiba Cardiovascular Center, Ichihara, Chiba 290-0512, Japan; Department of Cardiovascular Surgery, Chiba Cardiovascular Center, Ichihara, Chiba 290-0512, Japan; Department of Cardiovascular Surgery, Chiba Cardiovascular Center, Ichihara, Chiba 290-0512, Japan; Department of Cardiovascular Surgery, Chiba Cardiovascular Center, Ichihara, Chiba 290-0512, Japan

**Keywords:** sinus of Valsalva aneurysm, single coronary artery, Bentall procedure, aortic root replacement

## Abstract

We report a case of a sinus of Valsalva aneurysm associated with a single coronary artery, incidentally detected in a 73-year-old woman. Imaging revealed a Lipton type L2-A single coronary artery and a sinus of Valsalva aneurysm. The patient underwent successful Bentall surgery with careful coronary reimplantation and combined antegrade-retrograde myocardial protection. This case highlights surgical considerations in managing this uncommon combination of anomalies.

**Clinical registration number:** Because this was a single-patient case report and written informed consent for participation in medical research was obtained from the patient, institutional review board approval was waived in accordance with institutional policy.

## INTRODUCTION

The coexistence of a sinus of Valsalva aneurysm and a single coronary artery is extremely rare, with only a limited number of cases reported in the literature. We report a case of a sinus of Valsalva aneurysm associated with a single coronary artery that was incidentally detected during a routine health examination and successfully treated with Bentall surgery.

## CASE REPORT

A 73-year-old woman was referred to our institution for further evaluation after a heart murmur was detected during a health checkup. She had no significant past medical history, and her family history was negative for heritable aortic diseases or sudden death.

Transthoracic echocardiography on presentation showed a preserved left ventricular ejection fraction, moderate aortic regurgitation with a central jet, and an aneurysm of the sinus of Valsalva.

**Figure 1.**
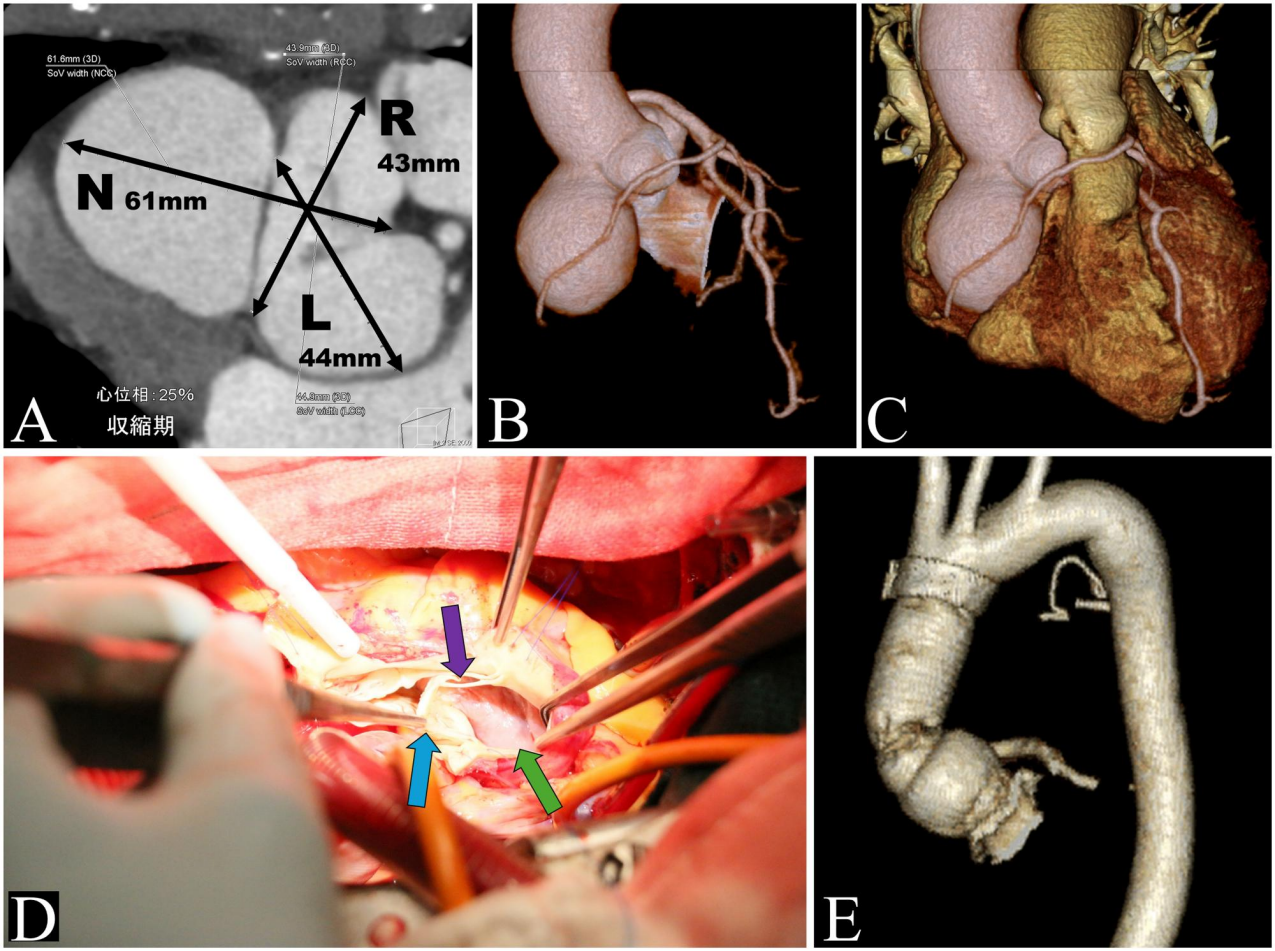
Preoperative Imaging, Intraoperative Findings, and Postoperative Computed Tomography. (A) Preoperative contrast-enhanced computed tomography at the level of the sinus of Valsalva demonstrates a 61-mm aneurysm of the non-coronary sinus. (B) Preoperative 3-dimensional reconstructed computed tomography image. (C) Anatomical findings of a single coronary artery in which the right coronary artery originates from the left main trunk and courses anterior to the pulmonary artery. (D) View of the aortic root during inspection. The non-coronary sinus side is being observed. Blue arrow: non-coronary cusp. Green arrow: The inner wall of the sinus of Valsalva, which is thinned and appears pale pink. Purple arrow: fenestration of the valve cusp. (E) Three-dimensional reconstruction image from postoperative contrast-enhanced computed tomography. Abbreviations: L, left coronary sinus; N, non-coronary sinus; R, right coronary sinus

Based on these findings, contrast-enhanced computed tomography was performed, which revealed a 61-mm sinus of Valsalva aneurysm protruding predominantly towards the non-coronary sinus (Figure 1A). In addition to the left anterior descending and circumflex arteries, the right coronary artery originated from the left main trunk and coursed anterior to the pulmonary artery (Figure 1B and C). According to the Lipton classification, the anomaly was diagnosed as an L2-A type single coronary artery.

Given the aneurysm size, surgical intervention with aortic root replacement was indicated. Through a median sternotomy, cardiopulmonary bypass was established via ascending aortic cannulation and right atrial drainage. After aortic cross-clamping, cardiac arrest was achieved using both antegrade and retrograde cardioplegia. Aortotomy revealed a markedly thinned and aneurysmal sinus of Valsalva (Figure 1D).

A composite graft consisting of a prosthetic graft and a bioprosthetic valve was used to reconstruct the aortic root. The single coronary ostium was carefully mobilized and reimplanted using the Carrel patch technique. During coronary reconstruction, a Nelaton catheter was inserted into the coronary orifice to maintain the lumen. Weaning from cardiopulmonary bypass was uneventful. Although discharge planning required additional time due to social circumstances, the postoperative course was uneventful and the patient was discharged on postoperative day 21. Postoperative contrast-enhanced computed tomography showed no abnormalities (Figure 1E). Histopathological examination of the resected aortic wall demonstrated cystic medial degeneration.

## DISCUSSION

A single coronary artery is an exceedingly rare congenital anomaly, with a reported incidence of 0.024% in the general population and representing only a small fraction of coronary artery anomalies.^1^ An interarterial course between the aorta and pulmonary artery, or an intraseptal course, is considered malignant and is associated with exertional myocardial ischaemia and sudden death.^2^

Sinus of Valsalva aneurysms have diverse aetiologies, although heritable aortic diseases are considered the most common underlying cause.^3^ In the literature, only 7 cases of combined single coronary artery and sinus of Valsalva aneurysm have been reported in English, including 4 ruptured and 3 unruptured aneurysms (Table 1). The causal relationship between these 2 anomalies remains unclear. There is limited evidence directly associating single coronary arteries with heritable aortic diseases, suggesting that the coexistence may be incidental.

**Table 1.** Previously Reported Cases of Valsalva Aneurysm Associated With a Single Coronary Artery

Case #	First author	Year	Sex	Age, years	Lipton type	Size of SVA, mm	Ruptured/unruptured	Pathology of SVA
1	Mohammad	2021	M	67	RIII	62	Unruptured	Not reported
2	Goto	1989	M	60	LII-P	Not reported	Ruptured	Not reported
3	Ishii	2003	M	26	LII-B or P	Not reported	Ruptured	Not reported
4	Chamsi-Pasha	1989	M	41	L	Not reported	Ruptured	Not reported
5	Dazai	1991	M	55	LI	Not reported	Unruptured	Infective endocarditis
6	Feldman	2005	M	26	LII-B	Not reported	Ruptured	Myxoid degeneration
7	Nishimura	2011	M	75	LI	46	Unruptured	Not reported
8	Mukasa	2025	F	73	LII-A	61	Unruptured	Cystic medial degeneration

Abbreviations: F, female; M, male; SVA, sinus of Valsalva aneurysm.

Several operative considerations are crucial in cases such as ours. First, careful dissection is required to avoid injury to the anomalous coronary artery. Second, secure reconstruction of the single coronary ostium is essential because the entire myocardial perfusion depends on a single arterial origin. We ensured precise reimplantation by inserting a Nelaton catheter into the coronary orifice during anastomosis. Third, myocardial protection must be optimized. In single coronary artery anatomy, myocardial perfusion patterns differ from those in normal coronary anatomy. Using either antegrade or retrograde cardioplegia alone may lead to inadequate regional protection; thus, both methods were combined to ensure uniform myocardial protection. The surgical technique of the Bentall procedure itself does not differ substantially from the standard approach. However, particular attention to the considerations described above is required.

## CONCLUSION

We present a rare case of a sinus of Valsalva aneurysm associated with a single coronary artery that was successfully treated with Bentall surgery. Although this combination is unusual, aortic root replacement can be performed safely when meticulous coronary reimplantation and comprehensive myocardial protection strategies are employed.

## Data Availability

The data that support the findings of this study are available from the corresponding author, Kotaro Mukasa, upon reasonable request. Some data may not be made available because of privacy or ethical restrictions.
